# Analysis of Codon Usage Bias in the Plastid Genome of *Diplandrorchis sinica* (Orchidaceae)

**DOI:** 10.3390/cimb46090582

**Published:** 2024-09-03

**Authors:** Xuhui Chen, Yudi Zhao, Shenghua Xu, Yingze Zhou, Lijie Zhang, Bo Qu, Yufeng Xu

**Affiliations:** 1 College of Bioscience and Biotechnology, Shenyang Agricultural University, Shenyang 110866, China; xhchen@syau.edu.cn (X.C.); 2021170211@stu.syau.edu.cn (S.X.); jct1005@stu.syau.edu.cn (Y.Z.); syau_qb@163.com (B.Q.); 2 College of Forestry, Shenyang Agricultural University, Shenyang 110866, China; zyd666@stu.syau.edu.cn (Y.Z.); zlj330@syau.edu.cn (L.Z.)

**Keywords:** *Diplandrorchis sinica*, saprophyte orchid, plastid genome, codon usage bias, Orchidaceae, phylogenetic evolution

## Abstract

In order to understand the bias and main affecting factors of codon usage in the plastid genome of *Diplandrorchis sinica*, which is a rare and endangered plant species in the Orchidaceae family, the complete plastid genome sequence of *D. sinica* was downloaded from the GenBank database and 20 protein-coding sequences that met the analysis requirements were finally selected. The GC content, length of the amino acid (Laa), relative synonymous codon usage (RSCU), and effective number of codon (ENC) of each gene and codon were calculated using the CodonW and EMBOSS online programs. Neutral plot analysis, ENC-plot analysis, PR2-plot analysis, and correspondence analysis were performed using Origin Pro 2024 software, and correlation analysis between various indicators was performed using SPSS 23.0 software. The results showed that the third base of the codon in the plastid genome of *D. sinica* was rich in A and T, with a GC_3_ content of 27%, which was lower than that of GC_1_ (45%) and GC_2_ (39%). The ENC value ranged from 35 to 57, with an average of 47. The codon usage bias was relatively low, and there was a significant positive correlation between ENC and GC_3_. There were a total of 32 codons with RSCU values greater than 1, of which 30 ended with either A or U. There were a total of nine optimal codons identified, namely, UCU, UCC, UCA, GCA, UUG, AUA, CGU, CGA, and GGU. This study indicated that the dominant factor affecting codon usage bias in the plastid genome of *D. sinica* was natural selection pressure, while the impact of base mutations was limited. The codon usage patterns were not closely related to gene types, and the distribution of photosynthetic system genes and ribosomal protein-coding gene loci was relatively scattered, indicating significant differences in the usage patterns of these gene codons. In addition, the codon usage patterns may not be related to whether the plant is a photosynthetic autotrophic or heterotrophic nutritional type. The results of this study could provide scientific references for the genomic evolution and phylogenetic research of plant species in the family Orchidaceae.

## 1. Introduction

*Diplandrorchis sinica* S. C. Chen is a relatively primitive relit herbaceous plant in the Orchidaceae family, named for its two fertile stamens located in the ventral and dorsalis direction of the tip of the stamen column. *D. sinica* is a small saprophytic plant with a height of only about 10 cm. The plant does not have any green leaves throughout its life and therefore cannot perform photosynthesis. Its roots are clusters of fleshy fibrous roots with upright, unbranched stems, terminal racemes, and pale green or greenish-white flowers [1]. The growth cycle of the plant is extremely short, only about 20 days, and the environmental requirements for its development and growth is high. This species was named and published by Chen S. C. in 1979 [1], who was a Chinese expert in orchid classification taxonomy, and is now accepted as a synonym of *Neottia gaudissartii* Hand.-Mazz. [2]. The type specimen was collected from Laotudingzi National Nature Reserve, Huanren County, Liaoning Province, and was once considered to be an endemic orchid in Liaoning Province and therefore received key protection [3]. At the same time, due to its extremely narrow distribution range and small population size, this species was also included in the first batch of China’s Rare and Endangered Protected Plants List [4], classified as critically endangered and receiving widespread attention from domestic and foreign scholars [5]. However, there have been no reports on the phylogeny, development, reproduction mode, and germplasm resource protection of this species so far due to the limitations of the species resources.

The plastid is a unique type of organelle in plants that are closely related to carbohydrate synthesis and storage, and it is also a semi-autonomous organelle with an independent genome that has autonomous replication, transcription, and translation functions [6]. The size, structure, GC content, functional gene composition, and gene arrangement order of the plastid genome are usually highly conserved. In most green plants, the plastid genome ranges from 140 to 160 kb in size, containing approximately 113 protein-coding genes and consisting of four parts forming a circular double-stranded structure [7]. However, there are still about 450 fungal heterotrophic plant species in nature which have no green leaves and rely entirely on mycorrhizal fungi to obtain all the organic matter they need [8]. It is generally believed that fungal heterotrophy is a unique survival strategy evolved by plants to adapt to the low-light living environments [9]. Studies have found that the plastid genomes of such plants are usually small, and due to the lack of photosynthetic capacity, the gene sequences related to photosynthesis in the plastids will gradually be lost or pseudogenized, and this degradation process is staged and irreversible [10,11,12]. Fungal heterotrophic plants are usually very sensitive to their living environment and are currently in an endangered stage that requires protection. Their unique survival skills have also attracted the attention of evolutionary biology and ecology researchers.

Codons are the basic units that transmit genetic information in organisms, serving as bridges and bonds between proteins and nucleic acids. The codons in living organisms are all triplet codons, meaning that each codon is composed of three consecutive bases, and the 64 codons encode a total of 20 basic amino acids and three termination codons. Among them, methionine and tryptophan are encoded by a unique codon, while the remaining amino acids are encoded by two to six different codons that can code for the same amino acid, which are called synonymous codons. In the genome of living organisms, certain synonymous codons may occur frequently, known as optimized codons, while certain codons appear less frequently or not at all, known as non-optimized codons, a phenomenon known as codon usage bias [13]. The phenomenon of codon usage bias is widely present in biological species and is the result of adaptation and selection in the long-term evolutionary history of species. Generally, due to the influence of natural selection pressure, the codon usage pattern exhibits species specificity [14,15]. Similarly, species with closer phylogenetic relationships or similar growth environments may have similar codon usage patterns [16]. Therefore, codon usage bias can reflect the environmental adaptation and molecular evolution of species to some extent [17]. Theoretical studies have predicted and experiments have shown that codon usage bias could lead to a higher efficiency of translation, and the usage of optimal codons can increase the rate of translation and hence affect the fitness of an organism [18,19]. Therefore, it also has certain prospects for genetic engineering applications, with preferred codons replacing unpreferred ones when conducting genetic engineering research [20,21].

In our previous work, we have sequenced, assembled, annotated, and analyzed the plasmid genome of *D. sinica*, submitted it to the GenBank database, and obtained the accession number MZ014629.1. On this basis, this study intends to use bioinformatics methods to further analyze the usage bias of the genome codons in the plastid genome of *D. sinica*, aiming to provide a reference for the study of the plastid evolution of *D. sinica* species.

## 2. Materials and Methods

### 2.1. Acquisition of Sequence Data

**Figure 1:**
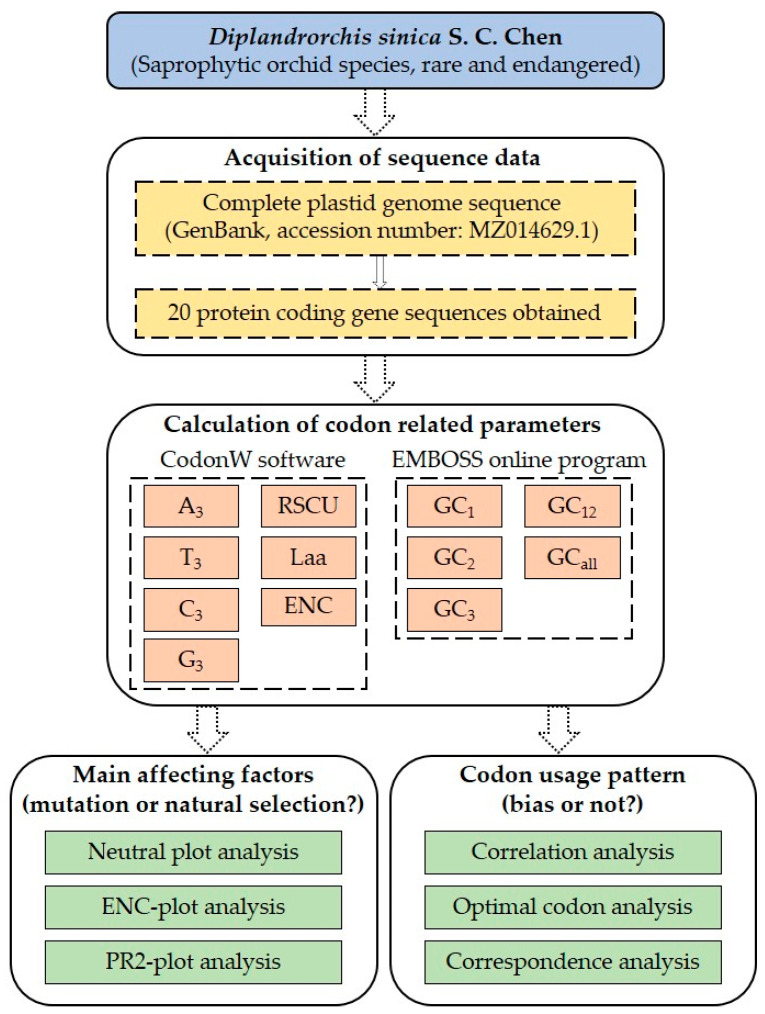
Flowchart for data acquisition and analysis of codon usage bias in the plastid genome of *Diplandrorchis sinica*.

The complete plasmid genome sequence of the *D. sinica* species was downloaded from the NCBI database, with a GenBank accession number of MZ014629.1. The sequence has a length of 109,435 bp, and a total of 55 protein-coding genes were identified. After removing duplicate gene sequences and sequences smaller than 300 bp, 20 protein-coding gene sequences were obtained that meet the requirements of subsequent bioinformatics analysis. The flowchart for the data acquisition and analysis of codon usage bias in the plastid genome of *D. sinica* is shown in Figure 1.

### 2.2. Calculation of Codon-Related Parameters

codonW 1.4 (https://galaxy.pasteur.fr/, accessed on 2 September 2024) was used to calculate the relative synonymous codon usage (RSCU), amino acid length (Laa), and effective number of codons (ENC) for each gene. ENC and RSCU can be used to measure the usage bias of synonymous codons. The ENC values range from 20 to 61, with larger values indicating weaker bias in codon usage and smaller values indicating stronger bias [22]. Generally, 45 is used as the critical point to distinguish codon bias. The RSCU is the ratio between the actual usage frequency and the theoretical usage frequency (1/number of codons coding for that amino acid) of a specific codon. When the RSCU < 1, it indicates that the actual usage frequency of the codon is lower than the theoretical use frequency. Meanwhile, when the RSCU > 1, it indicates that the actual usage frequency of the codon is higher than the theoretical usage frequency, and when the RSCU = 1, it indicates that there is no bias for the use of the codon [23].

Additionally, the contents of the third nucleotide T, C, A, and G in each gene codon were calculated and recorded as T_3_, C_3_, A_3_, and G_3_, respectively. The EMBOSS online program (http://www.bioinformatics.nl/emboss-explorer/, accessed on 2 September 2024) was used to calculate the overall GC content of each gene and recorded it as GC_all_. Furthermore, the GC contents at the first, second, and third positions of the codon in each gene were calculated and recorded as GC_1_, GC_2_, and GC_3_, respectively. Meanwhile, the average values of GC_1_ and GC_2_ for each gene codon were calculated and recorded as GC_12_, and the correlations between various parameters of these codons were analyzed.

### 2.3. Neutral Plot Analysis

Neutral plot analysis can be used to determine the factors that affect the codon usage bias. Using GC_3_ as the x-axis and GC_12_ as the y-axis for each gene, a scatter plot was drawn and linear regression was performed. The closer the scatter point is to the diagonal, the closer the values of GC_12_ and GC_3_ are, and the smaller the difference in the base composition of codons, indicating that codon usage bias is more influenced by mutations. On the contrary, there is a greater influence of selection pressure. The closer the regression coefficient is to 1, the higher the correlation between GC_12_ and GC_3_, and vice versa. The correlation analysis between GC_12_ and GC_3_ can be used to determine the main influencing factors of codon bias. If GC_12_ and GC_3_ are significantly correlated, it indicates that the base composition of GC_12_ and GC_3_ is similar, that is, mutation is the main influencing factor of codon usage bias. On the contrary, it indicates that there is a significant difference in the base composition between GC_12_ and GC_3_, indicating that natural selection is the main influencing factor of their codon usage bias [24].

### 2.4. ENC-Plot Analysis

ENC-plot analysis mainly uses image visualization methods to determine the influence of synonymous mutation on codon usage bias. The GC_3_ of each gene was taken as the x-axis and the ENC value was taken as the y-axis, and a scatter plot was drawn. Meanwhile, according to the formula ENC = 2 + GC_3_ + 29/[GC_3_^2^ + (1 − GC_3_)^2^], the theoretical values of ENC for each gene were calculated, and standard curves were plotted. If the scatter point is close to the standard curve, it indicates that codon usage bias is mainly affected by mutations, and if the scatter point is far from the standard curve, it is mainly affected by the selection pressure [25].

### 2.5. PR2-Plot Analysis

PR2-plot bias analysis can reflect the base composition of the third nucleotide in the codon. With G_3_/(G_3_ + C_3_) as the x-axis and A_3_/(A_3_ + T_3_) as the y-axis for each gene, a scatter plot was drawn. The center point in the figure represents the codon composition state under unbiased conditions, that is, A = T, G = C, while the distance and direction of each scatter point from the center point indicate the degree and direction of bias of the gene [26,27].

### 2.6. Optimal Codon Analysis

The optimal codon is determined based on the ENC value and RSCU value. The ENC values of all genes were sorted from small to large, and 10% from each end were selected to construct high and low bias libraries. The ΔRSCU values ≥ 0.08 in the library were defined as high-frequency codons, and the ΔRSCU values ≥ 0.08 and RSCU values ≥ 1 were defined as optimal codons [28].

### 2.7. Correspondence Analysis

Correspondence analysis can directly reflect the degree of similarity in codon usage bias between different genes. Based on the RSCU value of each codon, all genes were distributed in a multidimensional vector space, where the contribution rate of the first vector axis was the largest, and the contribution rate of the other axes decreased sequentially. A scatter plot was drawn, with the first vector axis as the horizontal axis and the second vector axis as the vertical axis, and the similarity degree of codon usage bias between different genes was determined based on the distance between them [29].

### 2.8. Statistical Analysis

The experimental data were analyzed and mapped using Origin Pro 2024 software. Data analysis was conducted using Pearson correlation analysis in SPSS 23.0 [30] to determine the correlation between various parameters of the codons.

## 3. Results

### 3.1. Composition Characteristics of Codons

A total of 20 genes in the plastid genome of *D. sinica* were selected for codon usage bias analysis after screening, with a total length of 22,151 bp, accounting for 20.24% of the total length of the plastid genome of *D. sinica*. The amino acid sequences encoded by these genes range in length from 100 to 2250, with an average length of 368. According to the statistical analysis of the codon composition characteristics of these genes, the average GC content was 37%. Among them, the contents of GC_1_, GC_2_, and GC_3_ were 45%, 39%, and 27%, respectively, with GC_1_ being greater than GC_2_ and greater than GC_3_, indicating that there are significant differences in the composition of bases at different positions, and they tend to end with A and T bases. The ENC values of each gene ranged from 35 to 57, with an average of 47. There were 13 genes with ENC values greater than 45, indicating a relatively weak usage bias in the plastid genome of *D. sinica* species (Table 1).

**Figure 2:**
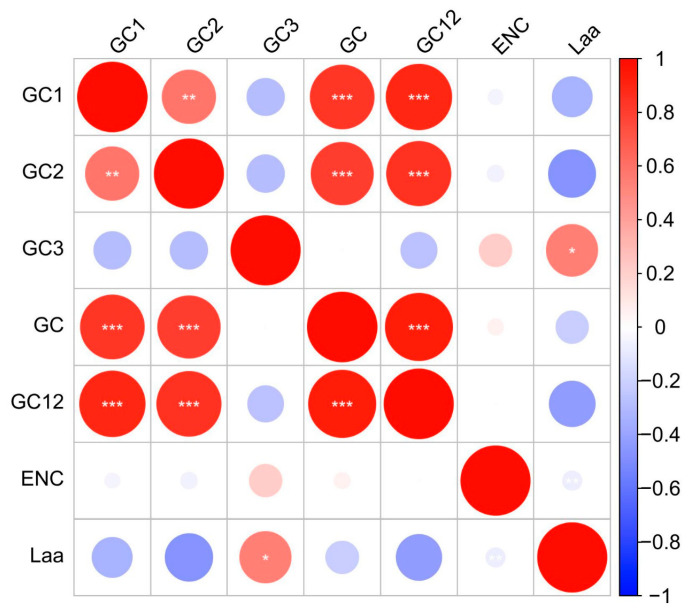
Correlation analysis between the indexes of codon usage in the plastid genome of *Diplandrorchis sinica.* Notes: “*” indicates a significant correlation at the *p* < 0.05 level, “**” indicates a significant correlation at the *p* < 0.01 level, “***” indicates a significant correlation at the *p* < 0.001 level.

Correlation analysis was conducted on various parameter indicators of gene codons (Figure 2), and the results showed that the correlation between GC_1_ and GC_2_ was extremely significant, but the correlation between GC_3_ and GC_1_, GC_2_, and GC_12_ was not significant, which indicated that the base compositions of the first and second positions of the gene codon of the encoding gene of the *D. sinica* plastid were relatively similar, while the base composition of the third position was relatively random and differed from the base compositions of the first and second positions. There was a significant correlation between GC and GC_1_ and GC_2_ and GC_12_, respectively, but no significant correlation between GC and GC_3_, indicating that the GC content was mainly determined by the first two bases. ENC was only significantly positively correlated with GC_3_, indicating that the base composition of the third position of the codon had a greater influence on the codon usage bias. The higher the GC content of the third position, the greater the ENC value and the weaker the codon usage bias. There was no significant correlation between ENC and Laa, indicating that codon usage bias was independent of the length of the coding gene sequence.

**Figure 3:**
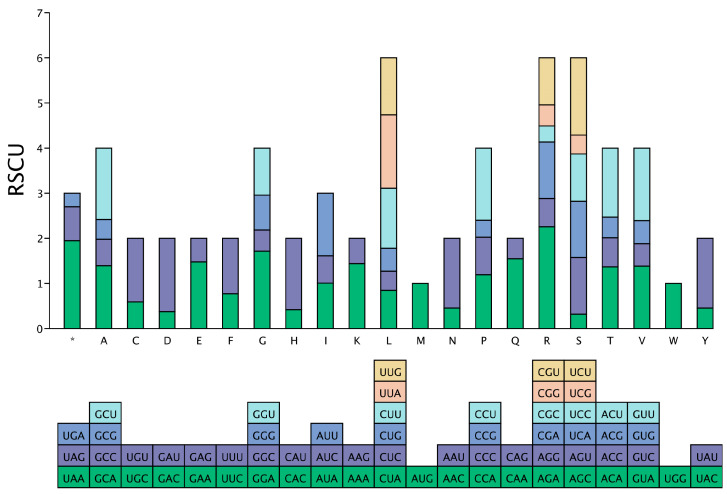
Relative synonymous codon usage (RSCU) analysis of genes in the plastid genome of *Diplandrorchis sinica*. Note: “*” stands for the termination codon.

The results of the RSCU analysis (Figure 3) showed that among the 62 codons of the 18 amino acids, excluding methionine (Met) and tryptophan (Trp), there were a total of 32 codons with RSCU values greater than 1, including 14 codons ending in A, 16 ending in U, 1 ending in G, and 1 ending in C. The results showed that the plastid genome of *D. sinica* tended to use synonymous codons ending in A and U, while codons with RSCU values less than 1 mostly end in C or G.

### 3.2. Neutral Plot Analysis

**Figure 4:**
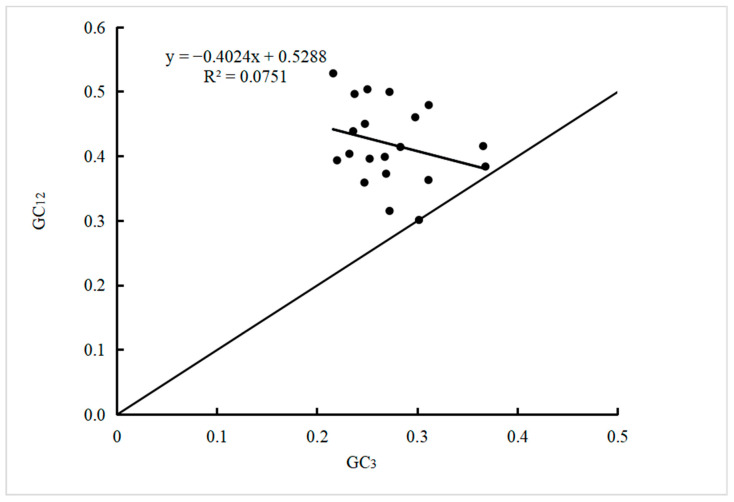
Analysis of neutrality plot.

The results of the neutral plot analysis (Figure 4) showed that the value of GC_12_ ranged from 0.30 and 0.54, and the value of GC_3_ ranged from 0.22 and 0.37. All gene loci were located above the diagonal of the midline, indicating significant differences in the base composition of the first, second, and third positions of the codon. The regression coefficient between GC_12_ and GC_3_ is −0.4024, with an absolute value far less than 1, indicating a very low degree of correlation between the two. Based on the correlation results between GC_12_ and GC_3_ in Table 2, it is indicated that the codon usage bias in the plastid genome of *D. sinica* is mainly affected by natural selection pressure.

### 3.3. ENC-Plot Analysis

**Figure 5:**
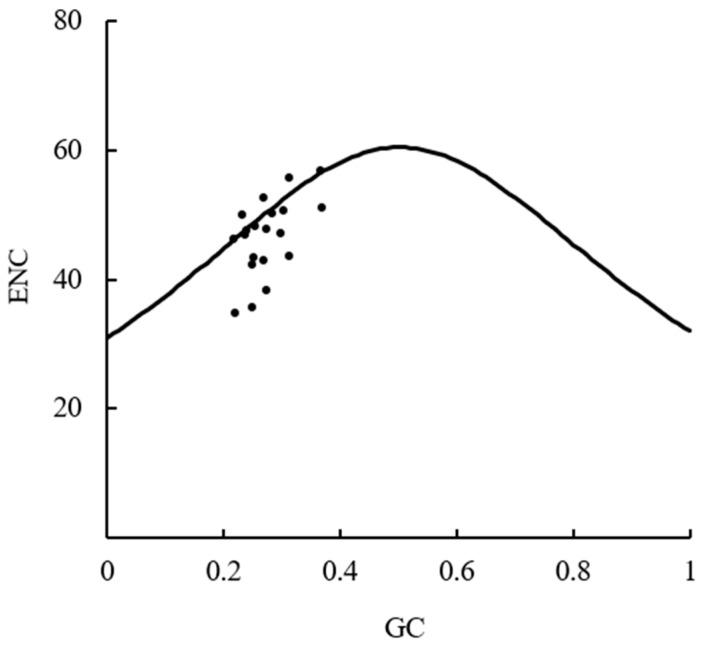
Analysis of ENC-plot.

The results of the ENC-plot analysis (Figure 5) showed that most of the gene loci fall below the standard curve but not on the standard curve, that is, there were differences between the actual ENC values and expected ENC values of most genes. This suggested that the selection pressure on the bias of codon usage in the plastid genome of *D. sinica* was greater than that of natural mutations.

### 3.4. PR2-Plot Analysis

**Figure 6:**
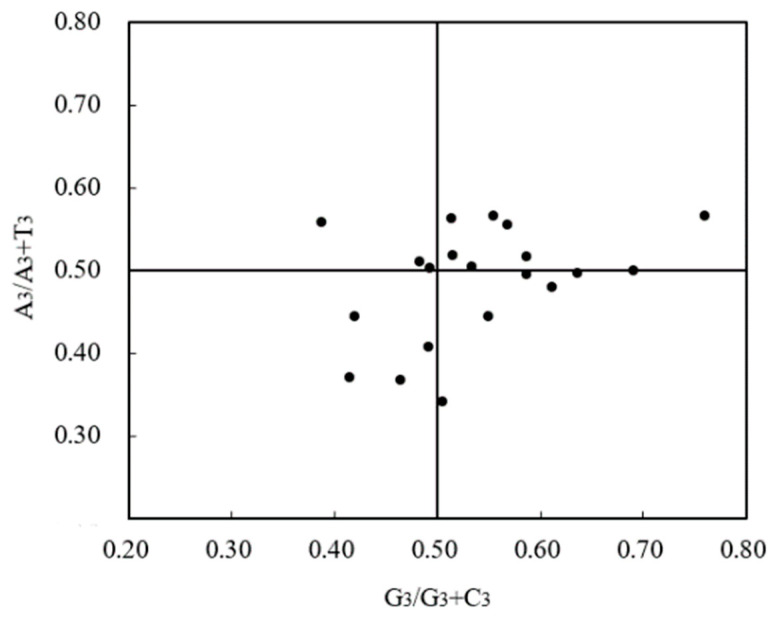
Analysis of PR2-plot.

The results of the PR2-plot analysis (Figure 6) showed that the distribution of gene loci in the four quadrants was not uniform, with more gene loci distributed in the upper right part of the PR2 plot, while the distribution of gene loci in the other three quadrants was relatively lesser, indicating a significant bias in the frequency of base usage at the third position of the codon, with A > T and G > C.

### 3.5. Optimal Codon Analysis

The RSCU and ΔRSCU values of each codon for high- and low-expression genes in the plastid genome of *D. sinica* were calculated, and a total of 23 codons were identified as high-expression codons, with ΔRSCU ≥ 0.08 as the standard. Among them, five codons ended in A, three codons ended in U, six codons ended in C, and eight codons ended in G (Table 2). Combined with the relative synonymous codon usage in the plastid genome of *D. sinica* (Figure 3), nine optimal codons were identified in the final analysis, namely, UCU, UCC, UCA, GCA, UUG, AUA, CGU, CGA, and GGU, of which four ended in A, three ended in U, one ended in G, and one ended in C (Table 2).

### 3.6. Correspondence Analysis

**Figure 7:**
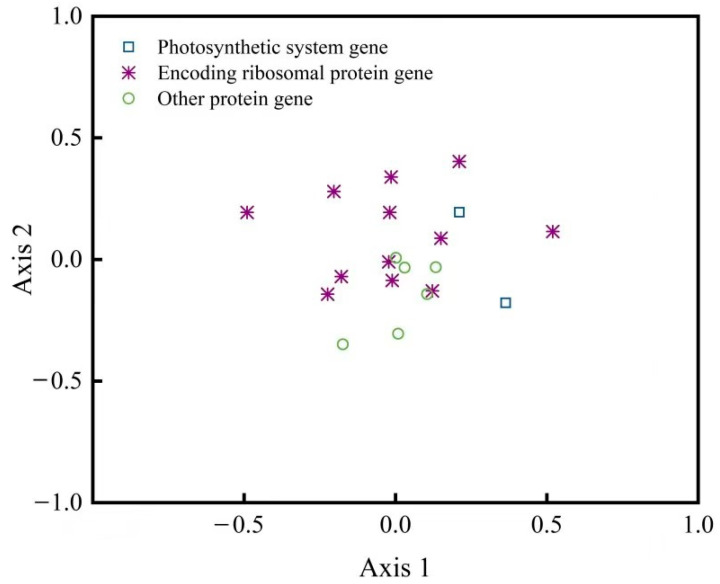
Corresponding analysis based on RSCU.

The results of the correspondence analysis showed that the RSCU of each gene codon in the plastid genome of *D. sinica* could be distributed in a 44-axis vector space, with the contribution rate of the first vector axis, second vector axis, third vector axis, and fourth vector axis being 12.63%, 11.06%, 8.43%, and 7.56%, respectively, and the cumulative difference contribution rate of the first four axes being 39.68%. Using the first and the second vector axes as horizontal and vertical axes, a scatter plot of each gene was plotted (Figure 7). It was found that the distribution of photosynthetic system genes and ribosomal protein-coding gene loci was relatively scattered, indicating that the usage patterns of these gene codons differed significantly.

## 4. Discussion

The degeneracy of codons can reduce harmful mutations and plays an important role in species stability. However, at the same time, organisms often form specific codon usage patterns during their long historical evolution process, namely, codon usage bias, which is of great significance for studying species evolution [28,31,32]. There are many indicators that can reflect the codon usage bias, such as RSCU, ENC, CAI (codon adaptation index), CBI (codon bias index), and FOP (frequency of optimal codons) [33], and research has shown that neutral plot analysis, ENC-plot analysis, PR2-plot analysis, and correspondence analysis could comprehensively reflect the codon usage bias of plastid and mitochondrial genomes, as well as the effects of natural selection and base mutations on codon usage bias [27,34]. In this study, we reported for the first time the bias for codon usage in the plastid genome of *D. sinica*, which is a rare and endangered orchid species. The results could provide evidence for the study of plastid evolution in saprophytic orchid species.

Because synonymous codon changes mainly occur at the third base of the codon, GC_3_ values are often used as the primary basis for measuring codon usage bias [35]. In this study, it was found that the base composition of the third position of the plastid encoding gene of *D. sinica* had a high degree of randomness, and the use frequency of A and T was higher than that of C and G, indicating a bias. This was consistent with the research results of plastid encoding genes of many green higher plant species, suggesting that the codon bias of higher plant plastid genomes ending in A and T is a relatively conservative characteristic [36,37,38]. It is speculated that this characteristic may not be related to whether the plant is a photosynthetic autotrophic or heterotrophic nutritional type. However, the use frequency of A was higher than that of T, and the use frequency of G was higher than that of C. This usage pattern is relatively rare in angiosperms [39] and is also different from the research results of T > A and G > C in plants of *Hemiptelea davidii* [40], Asteraceae [41] and 26 species of *Cymbidium* plants in the Orchidaceae family [42]. The results indicated that the codon usage bias of plant plastid genomes may be related to species, with significant differences between different species. At present, research on the codon usage bias of plastid genomes was mostly focused on photosynthetic autotrophic plant groups, while there were relatively few reports on the plastid genomes of saprophytic plants. Therefore, more evidence is needed to determine whether the usage pattern of plastid genome codons is related to the nutritional types of plant species.

It is generally believed that base mutations and natural selection are the main influencing factors of codon usage bias, and if there is a significant correlation between GC_3_ and GC_1_ or GC_2_ values, it indicates that codon usage bias is mainly affected by base mutations; otherwise, it is mainly dominated by natural selection [43]. Based on the results of correlation analysis, neutral plot analysis, and ENC-plot analysis, it can be seen that the use of codons in the plastid genome of *D. sinica* had a bias and was greatly affected by natural selection pressure, while being less affected by base mutations. This was consistent with the findings of *Cypripedium calceolus* [44] and 26 species of Juglandaceae [45]. However, the research results of different orchids were not the same. For example, the production of codon usage bias in the plastid genome of *Phalaenopsis* genus was affected by both base mutations and natural selection [46], while the formation of codon usage bias in the plastid genome of *Oncidium flexuosum* [37] and *Liparis bootanensis* [47] was more complex and may be the result of multiple factors working together. Therefore, although the phenomenon of biased codon usage in plant plastid genes is common, the dominant factors affecting its usage bias vary among different species, which may be related to the different evolutionary histories of each species.

In the process of protein translation, codons need to recognize each other, with tRNA carrying the corresponding anticodon in order to transfer free amino acid residues to the polypeptide chain, and the codons with the highest content of corresponding tRNA are called optimal codons [18]. The optimal codons can accelerate the translation speed by reducing the matching time with the corresponding tRNA [21,48]. Synonymous codons are used at different frequencies and therefore tend to form a large number of optimal codons [49]. In this study, nine optimal codons of the plastid genome of *D. sinica* were obtained, of which seven ended in A or T, consistent with the reported pattern of the optimal codons of the plastid genomes in most higher plants and algae being A or T and indicating that the evolution of plant plastid genomes is relatively conservative. The use of codons can affect the stability of mRNA and the efficiency of gene expression; therefore, codon optimization is crucial for molecular breeding work [50]. In future research, it may be considered to use optimal codons to improve the expression efficiency of genes of *D. sinica*, thereby guiding the biodiversity conservation of rare and endangered orchid germplasm resources.

Current research shows that codon usage preference can finely regulate gene expression at multiple levels. However, due to the relatively complex causes of codon usage bias, the current understanding of it is not yet deep, and many aspects still need to be further studied. In this study, the results of the correspondence analysis showed that codon usage patterns of different types of genes in the plastid genome of *D. sinica* were different. Different photosynthetic system genes and genes encoding ribosomal proteins exhibited different codon usage patterns, indicating significant differences in codon usage patterns among different genes. At present, the specific causes of this phenomenon and its biological significance for gene expression are not clear, and more molecular mechanisms research is needed.

## Figures and Tables

**Table 1:** The main parameters of codons for 20 protein-coding gene sequences in the plastid genome of *Diplandrorchis sinica*.

Genes	GC_1_	GC_2_	GC_3_	GC_all_	GC_12_	ENC	Laa
*accD*	0.38	0.34	0.25	0.32	0.36	42	497
*clpP*	0.59	0.37	0.31	0.42	0.48	56	195
*ndhB*	0.38	0.35	0.31	0.35	0.36	44	237
*ndhG*	0.32	0.29	0.30	0.30	0.30	51	135
*psaB*	0.40	0.43	0.37	0.40	0.42	57	237
*rbcL*	0.49	0.43	0.30	0.41	0.46	47	177
*rpl14*	0.51	0.37	0.24	0.37	0.44	47	122
*rpl16*	0.48	0.52	0.27	0.42	0.50	38	135
*rpl20*	0.37	0.37	0.27	0.34	0.37	53	133
*rpl22*	0.46	0.35	0.23	0.35	0.40	50	124
*rps11*	0.53	0.53	0.22	0.42	0.53	46	138
*rps12*	0.52	0.48	0.25	0.42	0.50	44	123
*rps14*	0.43	0.48	0.25	0.38	0.45	36	100
*rps2*	0.41	0.39	0.27	0.36	0.40	43	287
*rps3*	0.45	0.35	0.25	0.35	0.40	48	221
*rps4*	0.47	0.36	0.28	0.37	0.41	50	204
*rps7*	0.54	0.46	0.24	0.41	0.50	48	155
*rps8*	0.41	0.38	0.22	0.34	0.39	35	131
*ycf1*	0.36	0.27	0.27	0.30	0.32	48	1763
*ycf2*	0.42	0.35	0.37	0.38	0.38	51	2250
Average	0.45	0.39	0.27	0.37	0.42	47	368

**Table 2:** The optimal codons in the plastid genome of *Diplandrorchis sinica*.

Amino Acid	Codon	High Expressed Gene		Low Expressed Gene		ΔRSCU
		Number	RSCU	Number	RSCU	
S (Ser)	**UCU ^##^**	115	1.76	29	1.45	0.31
	**UCC** **^###^**	82	1.26	13	0.65	0.61
	**UCA** **^#^**	84	1.29	23	1.15	0.14
	UCG ^#^	30	0.46	4	0.2	0.26
	AGU	60	0.92	44	2.2	−1.28
	AGC	20	0.31	7	0.35	−0.04
P (Pro)	CCU	59	1.48	21	1.91	−0.43
	CCC	32	0.81	9	0.82	−0.01
	CCA	50	1.26	14	1.27	−0.01
	CCG ^##^	18	0.45	0	0	0.45
A (Ala)	GCU	42	1.5	28	2.15	−0.65
	GCC	18	0.64	9	0.69	−0.05
	**GCA** **^###^**	38	1.36	11	0.85	0.51
	GCG ^#^	14	0.5	4	0.31	0.19
L (Leu)	UUA	118	1.55	30	1.53	0.02
	**UUG** **^#^**	100	1.31	23	1.17	0.14
	CUU	94	1.23	31	1.58	−0.35
	CUC	34	0.45	9	0.46	−0.01
	CUA	69	0.91	19	0.97	−0.06
	CUG ^#^	42	0.55	6	0.31	0.24
I (Ile)	AUU	163	1.29	62	1.54	−0.25
	AUC	76	0.6	29	0.72	−0.12
	**AUA** **^##^**	141	1.11	30	0.74	0.37
V (Val)	GUU	67	1.61	27	1.54	0.07
	GUC ^##^	26	0.63	5	0.29	0.34
	GUA	46	1.11	32	1.83	−0.72
	GUG ^##^	27	0.65	6	0.34	0.31
N (Asn)	AAU	216	1.51	45	1.61	−0.10
	AAC ^#^	71	0.49	11	0.39	0.10
K (Lys)	AAA	288	1.42	35	1.52	−0.10
	AAG ^#^	117	0.58	11	0.48	0.10
Y (Tyr)	UAU	125	1.54	41	1.55	−0.01
	UAC	37	0.46	12	0.45	0.01
H (His)	CAU	82	1.53	20	1.74	−0.21
	CAC ^#^	25	0.47	3	0.26	0.21
Q (Gln)	CAA	141	1.54	29	1.49	0.05
	CAG	42	0.46	10	0.51	−0.05
T (Thr)	ACU	67	1.39	22	1.49	−0.10
	ACC	32	0.66	12	0.81	−0.15
	ACA	65	1.35	21	1.42	−0.07
	ACG ^##^	29	0.6	4	0.27	0.33
C (Cys)	UGU	39	1.34	16	1.6	−0.26
	UGC ^#^	19	0.66	4	0.4	0.26
F (Phe)	UUU	172	1.2	54	1.3	−0.10
	UUC ^#^	114	0.8	29	0.7	0.10
R (Arg)	**CGU** **^#^**	45	0.93	6	0.75	0.18
	CGC	18	0.37	4	0.5	−0.13
	**CGA** **^##^**	64	1.32	8	1	0.32
	CGG	26	0.54	4	0.5	0.04
	AGA	107	2.21	18	2.25	−0.04
	AGG	30	0.62	8	1	−0.38
* (TER)	UGA ^#^	2	1.2	0	0	1.20
	UAA	2	1.2	4	2.4	−1.20
	UAG	1	0.6	1	0.6	0
G (Gly)	**GGU** **^#^**	43	0.99	15	0.88	0.11
	GGC	15	0.34	12	0.71	−0.37
	GGA	76	1.75	33	1.94	−0.19
	GGG ^##^	40	0.92	8	0.47	0.45
D (Asp)	GAU	193	1.65	45	1.7	−0.05
	GAC	41	0.35	8	0.3	0.05
E (Glu)	GAA	203	1.41	49	1.48	−0.07
	GAG	84	0.59	17	0.52	0.07

Notes: the underlined codon indicates the genomic RSCU>1, “^#^” indicates ΔRSCU ≥ 0.08, “^##^” indicates ΔRSCU > 0.3, “^###^” indicates ΔRSCU > 0.5, the bold codons are the optimal codons, and “*” stands for the termination codon.

## Data Availability

The original contributions presented in the study are included in the article; further inquiries can be directed to the corresponding author.
